# Metallic Supports Accelerate Carbonization and Improve Morphological Stability of Polyacrylonitrile Nanofibers during Heat Treatment

**DOI:** 10.3390/ma14164686

**Published:** 2021-08-19

**Authors:** Jan Lukas Storck, Christian Hellert, Bennet Brockhagen, Martin Wortmann, Elise Diestelhorst, Natalie Frese, Timo Grothe, Andrea Ehrmann

**Affiliations:** 1 Faculty of Engineering and Mathematics, Bielefeld University of Applied Sciences, 33619 Bielefeld, Germany; jan_lukas.storck@fh-bielefeld.de (J.L.S.); christian.hellert@fh-bielefeld.de (C.H.); bennet.brockhagen@fh-bielefeld.de (B.B.); elise.diestelhorst@fh-bielefeld.de (E.D.); timo.grothe@fh-bielefeld.de (T.G.); 2 Faculty of Physics, Bielefeld University, 33615 Bielefeld, Germany; martin.wortmann@fh-bielefeld.de (M.W.); nfrese@uni-bielefeld.de (N.F.)

**Keywords:** electrospinning, stabilization, carbonization, metallic substrates, shrinkage, fiber morphology

## Abstract

Electrospun poly(acrylonitrile) (PAN) nanofibers are typical precursors of carbon nanofibers. During stabilization and carbonization, however, the morphology of pristine PAN nanofibers is not retained if the as-spun nanofiber mats are treated without an external mechanical force, since internal stress tends to relax, causing the whole mats to shrink significantly, while the individual fibers thicken and curl. Stretching the nanofiber mats during thermal treatment, in contrast, can result in fractures due to inhomogeneous stress. Previous studies have shown that stabilization and carbonization of PAN nanofibers electrospun on an aluminum substrate are efficient methods to retain the fiber mat dimensions without macroscopic cracks during heat treatment. In this work, we studied different procedures of mechanical fixation via metallic substrates during thermal treatment. The influence of the metallic substrate material as well as different methods of double-sided covering of the fibers, i.e., sandwiching, were investigated. The results revealed that sandwich configurations with double-sided metallic supports not only facilitate optimal preservation of the original fiber morphology but also significantly accelerate the carbonization process. It was found that unlike regularly carbonized nanofibers, the metal supports allow complete deoxygenation at low treatment temperature and that the obtained carbon nanofibers exhibit increased crystallinity.

## 1. Introduction

Carbon nanofibers are used in a broad range of applications, including composites—in which they offer improved mechanical properties [1,2,3]; energy storage devices—in which their electrochemical properties play an important role [4,5,6]; and filter applications—where their chemical and morphological properties are decisive [7,8,9]. Usually, carbon nanofibers are prepared by electrospinning of polymers, such as poly(acrylonitrile) (PAN), from solution or melt, followed by oxidative stabilization and high-temperature carbonization [10,11,12].

Both of these processes are achieved by thermal treatments. For stabilization, typically, a temperature of 280 °C is applied in air, approached by low heating rates in the order of 1 K/min, to perform oxidation, aromatization, cyclization, dehydrogenation, and crosslinking [13,14]. Afterward, the stabilized nanofibers can be carbonized at higher temperatures, typically above 500 °C, in a nitrogen atmosphere.

The resulting carbon nanofibers can show a broad range of chemical and morphological properties, depending not only on the carbonization temperature but also on the stabilization process. Considerable research has been focused on optimizing the process parameters of the stabilization step, such as the heating rate, terminal temperature, and duration of isothermal treatment [15,16,17].

Another rarely examined parameter, which can severely influence the nanofiber morphology and resulting mechanical properties, is the external mechanical force applied to the nanofiber mat during stabilization. Generally, thermal treatment results in the relaxation of internal tension, which results from severe elongation during electrospinning, thus causing undesired fiber deformations, which, in turn, result in macroscopic shrinkage of the nanofiber mats during this process [18,19,20]. This can be avoided to some extent by fixing the outer edges of the nanofiber mats or by straining aligned fiber bundles [21,22,23,24]. However, these approaches may lead to macroscopic fractures in the nanofiber mats due to the uneven force distribution. This is why previous studies focused on the possibility of stabilizing PAN nanofiber mats, electrospun onto aluminum foils or other substrates, which offer mechanical support during the first stages of heat treatment [25,26,27]. It was found that while stabilizing on a substrate could indeed partially prevent the nanofibers from undesired morphological changes, most substrate parameters did not significantly influence the resulting nanofibers. The heating rate was nevertheless found to play an important role in the chemical transition during stabilization. However, undesirable morphological changes of the nanofibers spun onto metallic substrates and stabilized as well as carbonized on these substrates were still observed.

Here, we investigated the hypothesis as to whether these morphological changes can be further reduced by double-sided metallic supports, i.e., sandwiching of the nanofibers during stabilization and carbonization. Therefore, a new approach was presented, comparing PAN nanofibers stabilized and incipiently carbonized with different support configurations after electrospinning onto an aluminum substrate. For comparative thermal treatment without support, the nanofiber mats were detached from the aluminum substrate. In addition to the one-sided support by the aluminum substrate, an aluminum sandwich (double-sided support) was investigated by placing another layer of aluminum on top of the nanofiber mat on the aluminum substrate. In addition, thermal treatment was conducted with a stainless steel sandwich (double-sided support), where the nanofiber mat was detached from the aluminum substrate and sandwiched between two stainless steel supports.

In a previous study [27], 500 °C was found to be ideal for obtaining straight nanofibers without significant morphological changes using aluminum substrates. In addition, morphological changes occur predominantly in the temperature range below 500 °C. Since aluminum with a melting temperature of 650 °C is not suitable for high-temperature carbonization, it is only used to bridge this critical temperature range. Further carbonization up to 1500 °C or even graphitization up to 2500 °C can, of course, be applied without a substrate in a subsequent step, if needed. This study, in line with earlier works, addresses the incipient carbonization in the critical temperature range up to 500 °C. 

## 2. Materials and Methods

The needleless industrial electrospinning machine Nanospider Lab (Elmarco, Liberec, Czech Republic) was used to prepare PAN nanofiber mats from 16% PAN (tradename X-PAN; Dralon, Dormagen, Germany), a co-polymer with 6% methyl-methacrylate, dissolved in dimethyl sulfoxide (DMSO, min. 99.9%; S3 Chemicals, Bad Oeynhausen, Germany) by stirring at ambient temperature for 2 h.

The following spinning parameters were chosen: voltage 80 kV, resulting current ~0.1 mA, nozzle diameter 0.9 mm, electrode–substrate distance 240 mm, carriage speed 100 mm/s, substrate speed 0 mm/min, relative humidity 32%, and temperature in the spinning chamber 22 °C. The spinning duration was chosen as 30 min. Spinning and solution parameters were identical to those applied in previous studies [26,27].

Aluminum foil (thickness 35 µm; Vireo.de, Merseburg, Germany) and nonwoven polypropylene (PP) (Elmarco) were used as substrates. In addition, stainless steel 1.4301 V2a sheets (thickness 500 µm; Stahlog GmbH, Hörselberg-Hainich, Germany) were used to sandwich the electrospun nanofiber mats during stabilization and carbonization. The sandwich configuration refers here to a support configuration for the heat treatment of a nanofiber mat with a double-sided metallic support.

After electrospinning, parts of the samples were stabilized in a B150 muffle oven (Nabertherm, Lilienthal, Germany) at a temperature of 280 °C for 1 h, approached with a heating rate of 0.25 K/min. A CTF 12/TZF 12 furnace (Carbolite Gero Ltd., Sheffield, UK) was subsequently used for incipient carbonization at 500 °C for 1 h, approached with a heating rate of 10 K/min in a nitrogen gas flow of 100 mL/min (STP).

In this way, the following samples were prepared:

PAN electrospun on a PP substrate (sample PP-E), stabilized (PP-S), and carbonized without a substrate (PP-C).

PAN electrospun on an aluminum substrate (sample AL-E), stabilized (AL-S), and carbonized (AL-C) on the aluminum substrate, which functions as a single-sided support. 

PAN electrospun on aluminum, detached, and afterward stabilized (Al-SW1-S) and carbonized (AL-SW1-C) with an aluminum sandwich configuration (double-sided metallic support).

PAN electrospun on aluminum, not detached, stabilized (AL-SW2-S) and carbonized (AL-SW2-C) with an aluminum sandwich configuration (double-sided metallic support; here, the nanofiber mat adheres to the bottom substrate of the sandwich configuration.

PAN electrospun on aluminum, detached, and afterward stabilized (STS-SW-S) and carbonized (STS-SW-C) with a stainless steel sandwich configuration (double-sided support).

Investigations of the fiber and mat morphologies were performed using a helium ion microscope (HIM) Orion Plus (Carl Zeiss, Jena, Germany) at 34.2 kV acceleration voltage. By defining the spot control as 6.5, a beam current of 0.1–0.2 pA was reached. The charging effects during secondary electron detection were compensated by using an electron flood gun after each line scan.

The areas of the nanofiber mats were investigated based on the evaluation of photographs with ImageJ (Software version 1.53e, 2021, National Institutes of Health, Bethesda, MD, USA). The images were taken from a fixed distance of 13 cm from the samples, wherein a ruler was positioned in plane with the samples. Using pixel measurement, an accurate scale was set for the images. The edges of the samples were enhanced by applying a threshold filter. The areas were determined using the analyze particles feature in ImageJ.

Fourier-transform infrared (FTIR) spectroscopy was performed in attenuated total reflection mode (ATR-FTIR), resulting in a penetration depth of about 1.7 µm, depending on the angle and wavenumber, i.e., less than 5% of the original thickness of the PAN nanofiber mats of about 50 µm. Spectra were taken from 4000 to 700 cm^−1^, averaged over 32 scans each and corrected for atmospheric noise.

Raman investigations were performed in backscattering mode using a LabRAM Aramis spectrometer (HORIBA Europe, Oberursel, Germany) with a cooled CCD detector and a helium-neon laser at 633 nm. The $I_{D}/I_{G}$ ratio was calculated using the peak amplitudes.

## 3. Results and Discussion

**Figure 1:**
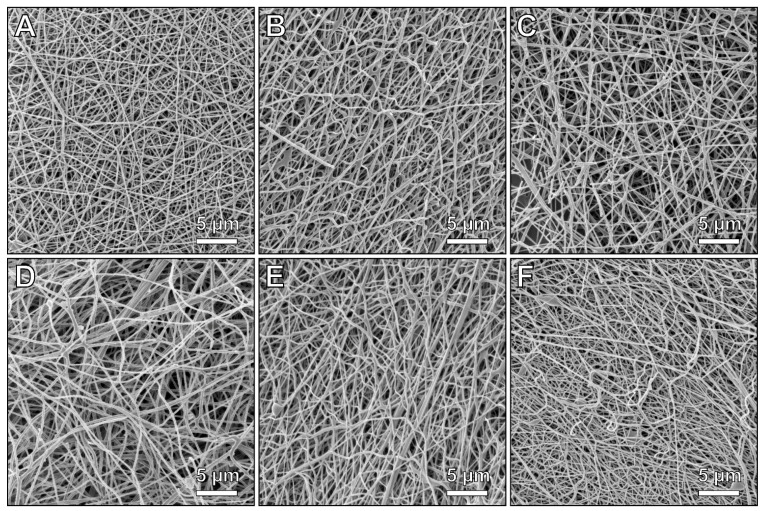
HIM images of PAN nanofibers with a field of view of (30 µm)^2^: (**A**) PP-E, (**B**) PP-C, (**C**) AL-C, (**D**) AL-SW1-C, (**E**) AL-SW2-C, and (**F**) STS-SW-C. Overview images with a field of view of (150 µm)^2^, including an image of sample AL-E, are shown in Figure S1 in the Supporting Information.

To examine the nanofiber morphology resulting from different support configurations, Figure 1 depicts HIM images of the PAN nanofibers after electrospinning as reference (Figure 1A), as well as all carbonized samples.

**Figure 2:**
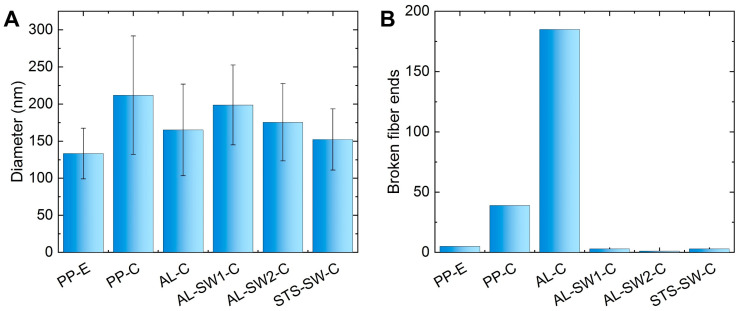
Evaluation of HIM images: (**A**) average diameters and (**B**) approximate numbers of broken fiber ends per image.

A comparison of the images showed that carbonization causes a more or less pronounced change in fiber morphology, depending on the support configuration, with more broken fibers and apparently larger fiber diameters, too. Figure 2 shows fiber diameters and the numbers of visible fiber ends per image, as depicted in Figure 1. It must be mentioned that in all cases, relatively small areas were investigated, not necessarily representing the whole nanofiber mat, so the relatively small amounts of visible broken fibers may quantitatively differ from other areas on the same sample, as is always the case in highly magnified images, showing only a few of the investigated features [28]. Figure S1 in the Supporting Information thus shows images with larger fields of view, including an image of sample AL-E electrospun on an aluminum substrate, which does not show a significant difference from sample PP-E electrospun on a PP substrate (Figure 1A). In these overview images, sample AL-SW1-C shows a more uneven morphology, as is also the case in Figure 1D.

Figure 2A shows the differences between average diameters of the original PAN nanofibers (PP-E) and the nanofibers carbonized in different support configurations. Here, it is visible that the largest nanofiber diameters could be found in sample PP-C, carbonized without metallic supports. Sandwiching a nanofiber mat, without adhesion to one of the outer metal substrates (due to electrospinning onto the substrate), did not clearly reduce the average fiber diameter in the case of an aluminum sandwich (AL-SW1-C), while sandwiching an unfixed nanofiber mat between stainless steel supports (STS-SW-C) resulted in a smaller fiber diameter. Electrospinning on an aluminum substrate, followed by stabilization and carbonization in this configuration (AL-C) or with an additional aluminum support in a sandwich configuration (AL-SW2-C), also helped to avoid a large increase in the fiber diameter. This confirms the previously [25,26,27] suggested solution for the production of relatively straight carbon nanofibers electrospun and heat-treated on aluminum substrates.

Figure 2B, however, illustrates a disadvantage of this method of stabilization and carbonization of a nanofiber mat fixed on an aluminum substrate. While the free relaxation during stabilization and carbonization (PP-C) already increased the amount of broken fiber ends per area, this number further increased strongly for stabilization and carbonization on an aluminum substrate (AL-C).

This problem was solved by sandwiching the fiber mats in between metallic supports during heat treatment. The number of broken fiber ends in all samples carbonized in those sandwiches was as low as in the original state. This demonstrates the advantage of the sandwich configuration instead of one-sided support by the substrate, thus mostly retaining the thin fiber diameter and avoiding fiber breakage.

It should be mentioned that X-ray fluorescence analysis of nanofibers carbonized on aluminum substrates was performed with a custom-made instrument and showed no evidence of transfer of metallic residues to the fibers. A Kα fluorescence signal from aluminum residues would have been expected at 1.486 keV. No signals were detected that were distinguishable from the background signal; thus, no thermally activated diffusion processes were found in the sandwiched nanofiber mats after carbonization.

**Figure 3:**
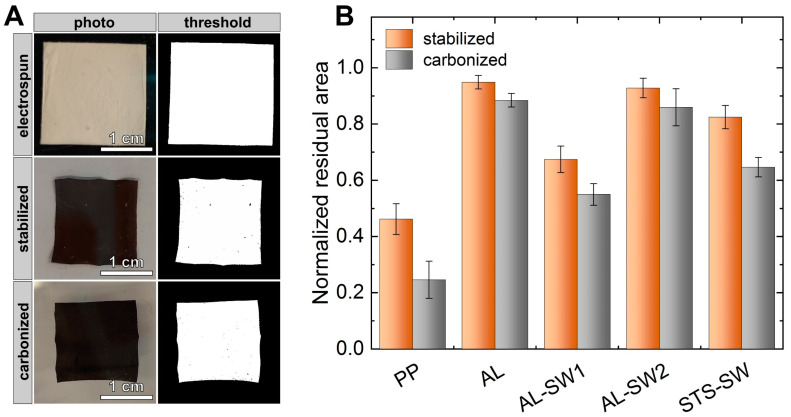
Area evaluation of heat-treated nanofiber mats: (**A**) exemplary procedure for the determination of the residual area and (**B**) normalized residual areas determined after stabilization and carbonization.

The macroscopic dimensions of the nanofiber mats were evaluated based on photographic images, as shown in Figure 3A. For this, five specimens of each sample of approx. (20 mm)^2^ each were examined. As the initial areas were not exactly the same, Figure 3 shows the normalized residual areas after stabilization and carbonization.

The samples heat-treated without support (PP) showed by far the smallest residual areas both after stabilization and after carbonization. The largest residual areas were observed for AL-C and AL-SW2-C, which were both adhered to an aluminum substrate during thermal treatment. The reason for this is that the adhesion at the metal–fiber interface mostly prohibits shrinkage of the nanofiber mat. 

**Figure 4:**
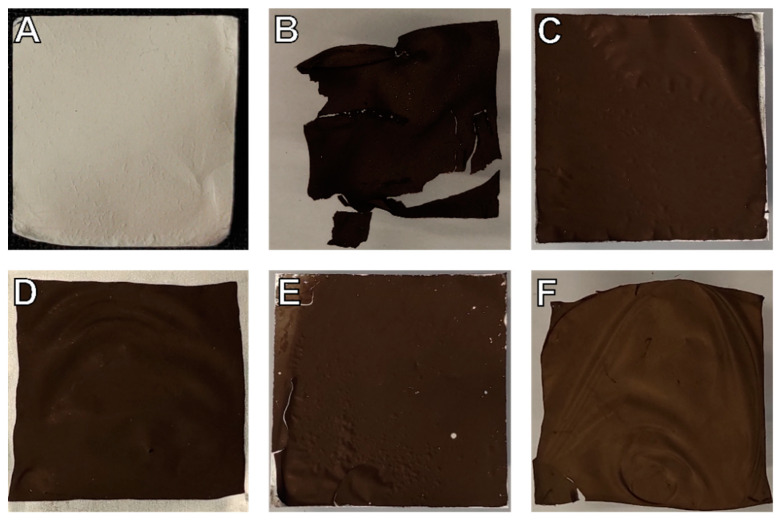
Exemplary photos of the samples: (**A**) PP-E, (**B**) PP-C, (**C**) AL-C, (**D**) AL-SW1-C, (**E**) AL-SW2-C, and (**F**) STS-SW-C.

Figure 4 illustrates further macroscopic morphological changes during heat treatment. The cracks and broken-off pieces of the sample carbonized without support (PP-C in Figure 4B) were particularly striking. Such damages can be significantly reduced by metallic supports. Moreover, the samples AL-SW1-C and STS-SW-C with detached nanofiber mats after electrospinning exhibited a more pronounced deformation and wrinkling compared to AL-C and AL-SW2-C, which results from the weaker adhesion due to the temporary detachment prior to the heat treatment in the sandwich configuration.

AL-SW2-C, i.e., nanofibers electrospun onto an aluminum substrate sandwiched with additional aluminum support on top (double-sided support), can be suggested as the ideal configuration for the production of carbon nanofibers with minimal morphological changes—both on a microscopic and on a macroscopic scale (cf. Figure 2B).

**Figure 5:**
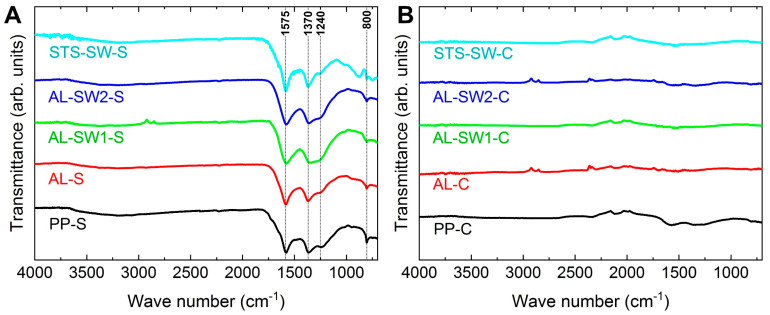
FTIR spectra of (**A**) stabilized and (**B**) carbonized nanofibers. The y-scales are identical in both diagrams, while the lines are vertically shifted for clarity.

Aside from the fiber morphology, the chemical composition of the carbonized nanofibers must be taken into account. Colorimetric measurements did not yield significant differences, which could unambiguously be attributed to the degree of stabilization or carbonization. For a more reliable evaluation of the degree of carbonization, the samples were examined by FTIR spectroscopy. Figure 5 depicts FTIR spectra of the nanofiber mats after stabilization and carbonization.

Generally, stabilized nanofibers show a spectrum with much more intense peaks than pristine or carbonized nanofibers (the former not shown here; see [21]). Thus, the height of the peaks could, in principle, be used as a measure of the degree of stabilization and carbonization, with carbonized nanofibers showing no peaks due to the chemical inertness of pure carbon [29,30]. 

Thus, FTIR spectra need to be evaluated differently. After stabilization, peaks can be expected at 800 cm^−1^, showing aromatic C−H vibrations after oxidative dehydrogenation aromatization [31]; at about 1575 cm^−1^, due to C=N and C=C stretching vibrations; at about 1370 cm^−1^, due to C–H bending and C–H_2_ wagging; and at about 1240 cm^–1^, due to oxygen crosslinking between the polymer chains, resulting in C–O vibrations [15]. Only the latter showed variations in the FTIR spectra of the stabilized nanofiber mats, with the largest C–O peak in sample AL-SW1-S and the smallest one in sample STS-SW-S, while the other characteristic peaks were quite similar in all spectra. This shows that, in general, double-sided supports in the sandwich configuration do not impair the stabilization result. However, further investigations are needed, as the metallic supports possibly prevent oxygen from reaching the fibers during stabilization. At about 2100 cm^−1^, some of the carbonized samples showed a common artifact, which is caused by the incompletely compensated strong absorption of the diamond ATR crystal.

After incipient carbonization, only sample PP-C showed residues of the characteristic peaks at 1575 and 1370 cm^−1^, indicating incomplete deoxygenation, which is common for a low carbonization temperature of 500 °C. However, the absence of peaks in all the other spectra indicates mostly completed deoxygenation, i.e., chemical inertness, for all samples that were at least partially covered by metal supports during heat treatment. The results suggest that metallic supports, possibly by catalytic activity at the interfaces, accelerate the decomposition reactions involved in the carbonization process. 

**Figure 6:**
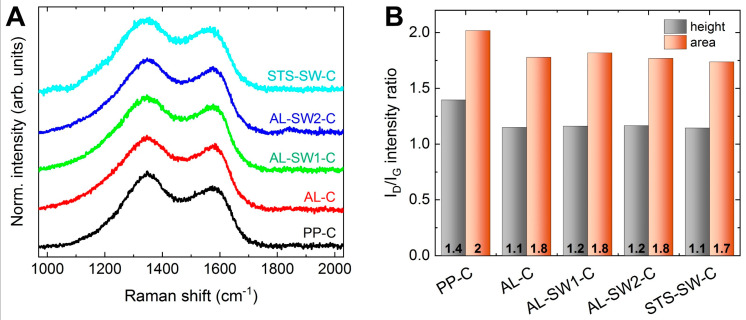
(**A**) D and G band regions in the Raman spectra of the carbonized nanofibers (lines are vertically shifted for clarity) and (**B**) the respective $I_{D}/I_{G}$ ratios determined by deconvolution (see Figure S2 in the Supporting Information) given both as a ratio of peak height and as a ratio of peak area. The Raman spectrum of as-spun PAN nanofibers is shown for comparison in Figure S3 in the Supporting Information.

Figure 6A shows the D and G bands in the Raman spectra of the carbonized nanofibers. The G band results from in-plane sp^2^ stretch vibrations in aromatic carbon. The D band is attributed to defects of bond angles, bond lengths, and hybridizations in a graphite lattice at the boundaries of crystalline domains. It is associated with semi-crystalline aromatic carbon structures, and it is not affected by functional groups or hydrogen bonds. Both an infinite graphite lattice and completely amorphous carbon show only a G band and no D band. The intensity ratio, i.e., the ratio of defective-to-ordered graphitic domains, is typically considered an important factor for the evaluation of crystallinity in carbonaceous materials. The intensity ratio was calculated both by peak area and by peak height (the latter being more commonly used) of the main D and G peaks of the deconvoluted spectral region, as shown in Figure S2 in the Supporting Information. A decreasing $I_{D}/I_{G}$ ratio indicates an increasing degree of crystallinity [32,33,34]. As seen in Figure 6B, the $I_{D}/I_{G}$ ratios showed similar values—around 1.2 by height (and 1.8 by area) for all samples—with the exception of PP-C, showing a notably higher value of 1.4 by height (and 2 by area), which underpins the interpretation of the FTIR spectra. It can be concluded that all samples that were at least partially covered with metal exhibited a higher degree of both carbonization and crystallinity.

## 4. Conclusions

Oxidative stabilization and incipient pyrolytic carbonization of PAN nanofibers were performed in different configurations with metallic supports: without fixation as a reference, adhered on an aluminum substrate on which the nanofibers were electrospun, as well as in different sandwich configurations, with the nanofiber mats sandwiched between two metal supports. By stabilizing and carbonizing in a sandwich with a double-sided metallic support, the original fiber morphology was retained and even the carbonization process was accelerated, allowing for complete deoxygenation at low carbonization temperature and increasing crystallinity of the resulting carbon nanofibers. The proposed procedure thus allows preserving the desired fiber morphology in the critical temperature range in which morphological changes usually occur. These high-quality fibers can then be graphitized at higher temperatures, if needed.

## Data Availability

All data produced in this study are presented in this paper.

## Supplementary Materials

The following are available online at https://www.mdpi.com/article/10.3390/ma14164686/s1, Figure S1. HIM images of the same samples, as shown in Figure 1, in the paper with a field of view of (150 µm)²; Figure S2. (A) D and G band region in the Raman spectra of carbonized nanofibers (lines are vertically shifted for clarity) and (B) numerical values of the deconvolution shown in (A); Figure S3. Raman spectrum of as-spun PAN nanofibers.
