# Supplementary material for: Metallic Supports Accelerate Carbonization and Improve Morphological Stability of Polyacrylonitrile Nanofibers during Heat Treatment
Source: Materials (Basel). 2021 Aug 19;14(16):4686. doi: 10.3390/ma14164686 (PMC8401406; doi:10.3390/ma14164686)
Supplement: Supplementary file 1 [file materials-14-04686-s001.zip › materials-1330652-supplementary.pdf]

materials

MDPI

Article

Metallic Supports Accelerate Carbonization and Improve Morphological Stability of Polyacrylonitrile Nanofibers during Heat Treatment

Jan Lukas Storck [1], Christian Hellert [1], Bennet Brockhagen [1], Martin Wortmann [2], Elise Diestelhorst [1], Natalie Frese [2], Timo Grothe [1] and Andrea Ehrmann [1,*]

[1] Faculty of Engineering and Mathematics, Bielefeld University of Applied Sciences, 33619 Bielefeld, Germany; jan_lukas.storck@fh-bielefeld.de (J.L.S.); christian.hellert@fh-bielefeld.de (C.H.); bennet.brockhagen@fh-bielefeld.de (B.B.); elise.diestelhorst@fh-bielefeld.de (E.D.); timo.grothe@fh-bielefeld.de (T.G.)

[2] Faculty of Physics, Bielefeld University, 33615 Bielefeld, Germany; martin.wortmann@fh-bielefeld.de (M.W.); nfrese@uni-bielefeld.de (N.F.)

* Correspondence: andrea.ehrmann@fh-bielefeld.de

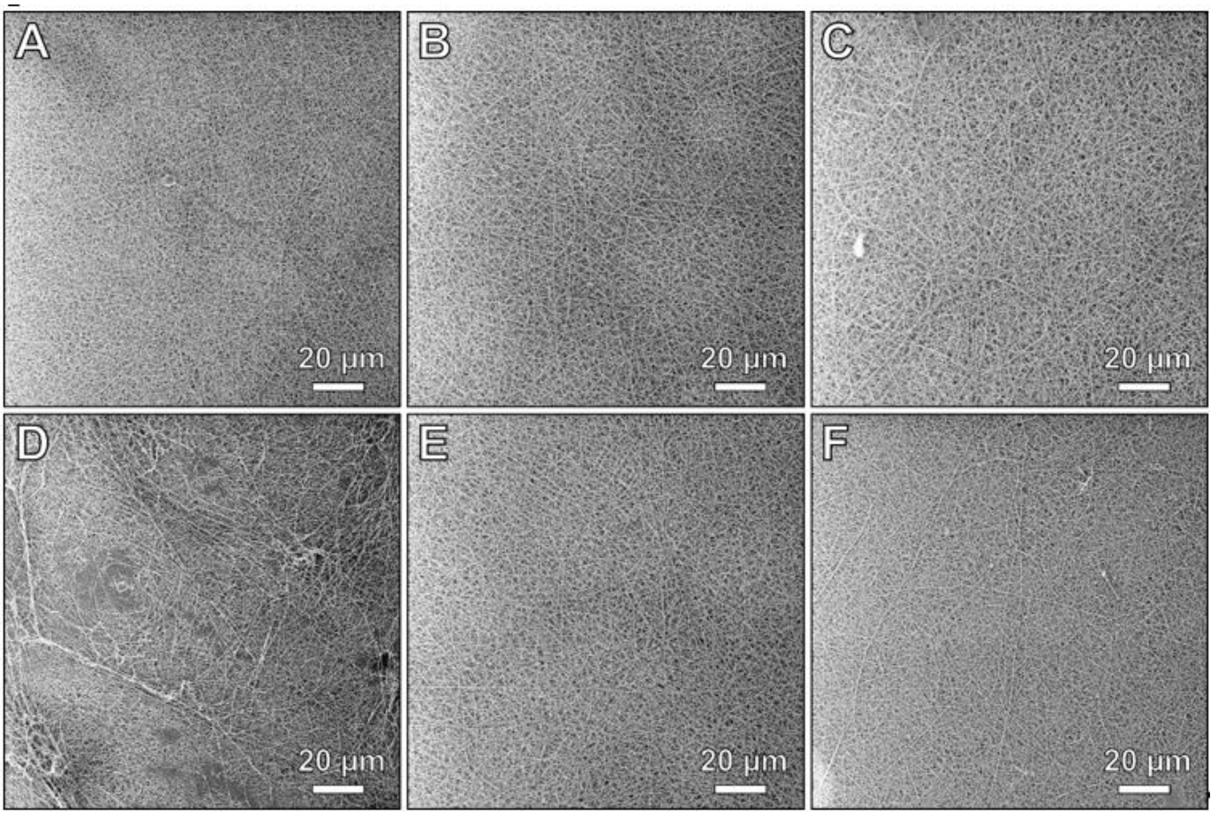

Figure S1. HIM images of the same samples as shown in Figure 1 in the paper with a field of view of (150 µm)^2: (**A**) AL-E; (**B**) PP-C; (**c**) AL-C; (**D**) AL-SW1-C; (**E**) AL-SW2-C and (**F**) STS-SW-C.

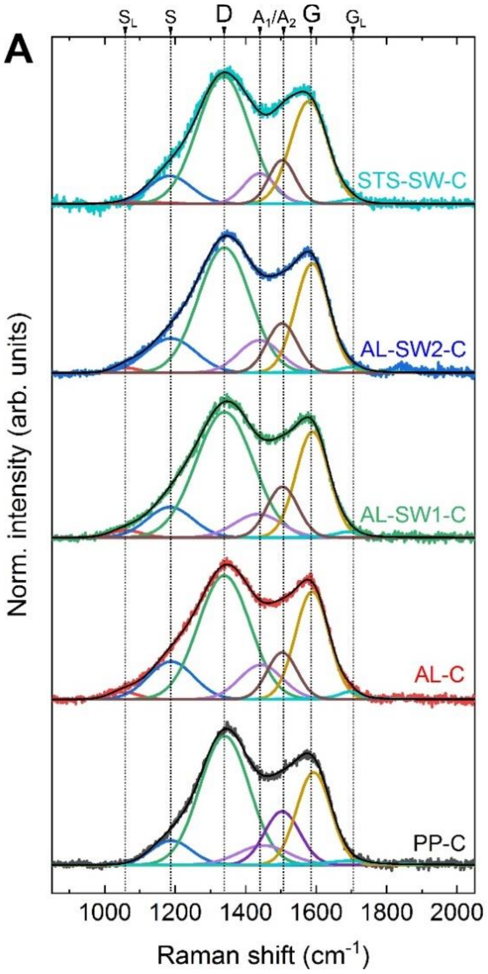

B

peak		norm. peak area (a.u.)	FWHM (cm^{-1})	max height (a.u.)	peak center (cm^{-1})
	PP-C	0.1	111.1	0.0	1056.0
	AL-C	5.2	98.2	0.1	1053.6
S_L	AL-SW1-C	5.5	109.5	0.1	1059.5
	AL-SW2-C	3.2	84.5	0.0	1060.8
	STS-SW-C	2.0	166.0	0.0	1075.3
	PP-C	23.4	135.2	0.2	1188.9
	AL-C	41.2	152.3	0.3	1188.5
S	AL-SW1-C	41.6	151.0	0.3	1194.0
	AL-SW2-C	40.9	166.3	0.2	1187.7
	STS-SW-C	28.5	142.8	0.2	1185.0
	PP-C	152.7	165.9	0.9	1340.3
	AL-C	153.9	173.9	0.8	1339.0
D	AL-SW1-C	155.1	171.9	0.9	1340.1
	AL-SW2-C	155.6	173.9	0.8	1339.5
	STS-SW-C	158.5	173.6	0.9	1336.9
	PP-C	23.5	169.0	0.1	1450.4
	AL-C	35.4	143.6	0.2	1444.0
A_1	AL-SW1-C	35.4	154.6	0.2	1443.3
	AL-SW2-C	32.5	140.1	0.2	1439.4
	STS-SW-C	23.3	107.3	0.2	1440.0
	PP-C	45.3	118.4	0.4	1503.7
	AL-C	33.6	100.3	0.3	1503.8
A_2	AL-SW1-C	37.7	103.8	0.3	1504.8
	AL-SW2-C	36.1	102.3	0.3	1503.5
	STS-SW-C	27.6	88.7	0.3	1503.6
	PP-C	75.7	114.5	0.6	1593.3
	AL-C	86.5	112.4	0.7	1588.9
G	AL-SW1-C	85.3	111.6	0.7	1589.9
	AL-SW2-C	87.9	112.5	0.7	1589.1
	STS-SW-C	91.2	124.1	0.7	1580.3
	PP-C	6.3	143.1	0.0	1695.3
	AL-C	5.9	73.2	0.1	1694.1
G_L	AL-SW1-C	5.6	91.7	0.0	1690.0
	AL-SW2-C	5.3	88.2	0.0	1699.0
	STS-SW-C	3.5	75.9	0.0	1696.3

Figure S2. (**A**) D and G band region in the Raman spectra of the carbonized nanofibers (lines are vertically shifted for clarity) and (**B**) numerical values of the deconvolution shown in A. While the D and G bands account for the largest portion of the signal intensity in this spectral region, several intermediate peaks arise upon deconvolution. Their distinction, designation, and significance are controversial. The deconvolution has only been used to evaluate accurately the D and G band contribution.

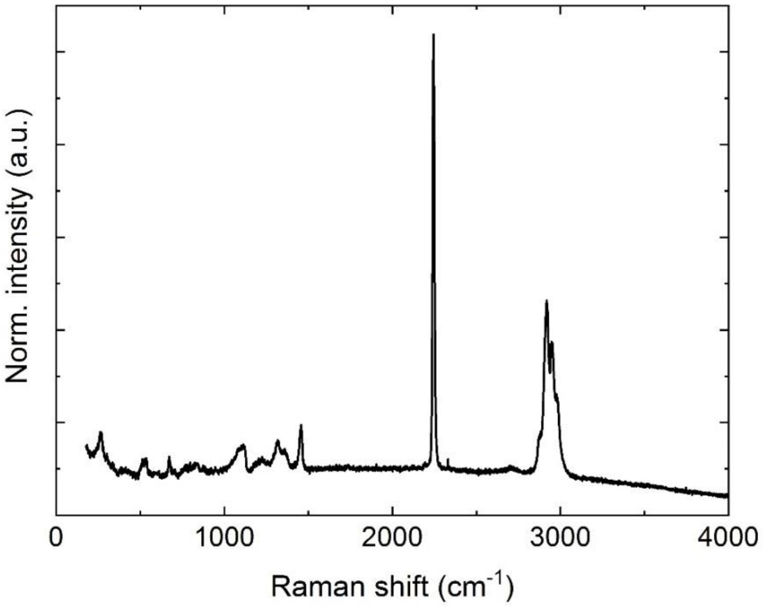

Figure S3. Raman spectrum of as-spun PAN nanofibers.
